# The Association of Epileptic Seizures after Acute Ischemic Stroke with Cerebral Cortical Involvement and Electroencephalographic Changes

**DOI:** 10.3390/medicina60050768

**Published:** 2024-05-06

**Authors:** Agnė Šmigelskytė, Gabija Rimkuvienė, Dominyka Žukaitė, Gerta Repečkaitė, Giedrė Jurkevičienė

**Affiliations:** 1 Department of Neurology, Lithuanian University of Health Sciences, A. Mickevičiaus Str. 9, LT-44307 Kaunas, Lithuania; 2 Department of Radiology, Lithuanian University of Health Sciences, A. Mickevičiaus Str. 9, LT-44307 Kaunas, Lithuania

**Keywords:** epileptic seizures, early epileptic seizures, late epileptic seizures, epilepsy, post-stroke epilepsy, stroke, ischemic stroke, electroencephalogram

## Abstract

*Background and objectives*: while acute ischemic stroke is the leading cause of epilepsy in the elderly population, data about its risk factors have been conflicting. Therefore, the aim of our study is to determine the association of early and late epileptic seizures after acute ischemic stroke with cerebral cortical involvement and electroencephalographic changes. *Materials and methods*: a prospective cohort study in the Hospital of the Lithuanian University of Health Sciences Kaunas Clinics Department of Neurology was conducted and enrolled 376 acute ischemic stroke patients. Data about the demographical, clinical, radiological, and encephalographic changes was gathered. Patients were followed for 1 year after stroke and assessed for late ES. *Results*: the incidence of ES was 4.5%, the incidence of early ES was 2.7% and the incidence of late ES was 2.4%. The occurrence of early ES increased the probability of developing late ES. There was no association between acute cerebral cortical damage and the occurrence of ES, including both early and late ES. However, interictal epileptiform discharges were associated with the occurrence of ES, including both early and late ES.

## 1. Introduction

Acute ischemic stroke is the leading cause of epilepsy in the elderly population. Due to advances in stroke treatment methods and their accessibility, patients have better stroke outcomes. However, in an aging population, post-stroke epilepsy remains a growing healthcare concern. According to some studies, strokes account for 21–50% of newly diagnosed epilepsies in adults, followed by brain tumors 4–16% and dementia 2–18% [1,2].

When epileptic seizures (ES) occur after a stroke, they are divided into early and late ES. Early ESs are considered to be acute symptomatic seizures due to biochemical reactions in the ischemic brain tissue. Meanwhile, late ESs occur as a result of a structural change in the brain with neuronal hyperexcitability and altered neuronal networks [3,4,5]. Therefore, according to the latest definition by Ilea, post-stroke epilepsy could be diagnosed after a single late ES [6]. Although a single early ES does not constitute the diagnosis of epilepsy, it increases the risk of late ES [7]. The time cut-off point between early and late ES is somewhat arbitrary and is most commonly considered to be 7 days [8].

The clinical significance of early and late ES after acute ischemic stroke is relatively well documented. The occurrence of ES leads to longer hospital stays, reduced functional independence, higher incidence of dementia, and death [9,10,11,12,13]. This suggests that patients at risk for ES require more care, additional follow-up and preventative measures, and should be recognized as soon as possible. In order to identify patients at risk of ES and the aforementioned complications, various scores have been developed, the most commonly utilized being the SeLECT score [14]. Machine-learning models have also been built [15]. However, with insufficient data on which risk factors have the biggest impact on ES incidence, current prognostic models are limited and require further optimization.

Data about demographic and clinical risk factors for ES after acute ischemic stroke have been conflicting. Some studies link younger age [16,17,18], atrial fibrillation [19,20], hypertension [21], large-artery atherosclerosis [22], and stroke severity [16,23] with an increased risk of ES, while others fail to produce similar results [7,24]. Stroke characteristics, identifiable with brain imaging, have also been investigated. For example, ischemic findings in the middle cerebral artery [17] and the posterior cerebral artery [22] territories have been associated with ES. Furthermore, cerebral cortical involvement is regularly described as a risk factor for ES by various authors [24,25].

Electroencephalogram (EEG) changes during the acute period of ischemic stroke remain understudied. According to some authors, epileptiform abnormalities might predict ES [26,27]. However, the time frame of when EEG findings might be associated with ES remains undetermined. Usually, researchers attempt to record an EEG during the first 72 h after stroke, which would be a challenge in some hospitals. Understandably, some patients would benefit from a more widespread use of EEG in acute ischemic stroke cases, especially in detecting non-convulsive ES [28].

Due to the contradictory findings described in the literature, the aim of our study was to determine the association of early and late ES after acute ischemic stroke with cerebral cortical involvement and electroencephalographic changes.

## 2. Materials and Methods

### 2.1. Study Design

We conducted a prospective cohort study in the Hospital of the Lithuanian University of Health Sciences Kaunas Clinics Department of Neurology from 1 January 2022 to 31 December 2023.

Patients were enrolled in the study during the first few days of hospitalization due to acute ischemic stroke. We collected their demographical and clinical data. A radiologist reevaluated brain imaging (CT or MRI) for cortical damage. When possible, a routine EEG was recorded. ESs were reported by relatives, medical staff, or the patients themselves. Early ESs were considered those that occurred during the first week (≤7 days) after acute ischemic stroke [8]. Patients were followed for 1 year after stroke and assessed for late ES.

This study was approved by the Kaunas Regional Biomedical Research Ethics Committee (P1-BE-2-38/2022).

### 2.2. Selection of Patients

The population of this study consisted of adult patients (≥18 years) hospitalized with clinical symptoms of acute ischemic stroke and acute ischemic findings on a brain CT or MRI. Patients with previously documented or concurrent head trauma; intracerebral, subarachnoid, or epidural hemorrhages; brain or vessel malformations; primary or secondary tumors of the central nervous system; and diagnosis of epilepsy prior to stroke were not included.

By using the latest epidemiological data on strokes in Lithuania [29] and Cochran‘s formula (confidence level 95%, precision level 5%), we determined the required sample size for our study to be 376 acute ischemic stroke patients. In total, 417 patients with clinically suspected acute ischemic stroke or their relatives (in cases of aphasia or coma) gave written consent to participate in the study. However, in 21 cases, no radiological findings indicative of ischemic stroke were observed, or the patients were diagnosed with other conditions and, as such, excluded from the study. In 14 patients, ischemic stroke was complicated by hemorrhagic transformation, and they had to be excluded. Seeing as it is an independent risk factor of ES [30,31], we intended to evaluate the effects of ischemic lesions only. During the observation period, 5 patients were treated for recurrent stroke in other hospitals. Since we could not access their data, they were excluded from the study. Additionally, 1 patient suffered a severe head injury and was also excluded. Therefore, the final sample size reached the calculated target of 376 acute ischemic stroke patients.

### 2.3. EEG Registration

We were able to record a routine EEG with video in 84 (22.3%) patients, while others could not be evaluated due to technical constraints (restricted equipment availability for research purposes and transportation difficulties of severely ill patients). No patients received antiepileptic drugs prior to EEG registration. We aimed to register the EEG as soon as possible after acute ischemic stroke. The median time of EEG registration was 3 (range 1–10) days. Sixty-two (73.8%) EEGs were registered during the first 3 days after stroke, and twenty-two (26.2%) EEGs were registered after 3 days. EEG electrodes were placed according to the international 10–20 system, and the recording was 30 min long.

### 2.4. Follow-Up

To document cases of late ES, we contacted patients or their relatives over the phone 3, 6, and 12 months after ischemic stroke. Out of 376 patients, 290 (77.1%) were successfully contacted after 3 months, 220 (58.5%) after 6 months, and 127 (33.8%) after 12 months.

### 2.5. Classification of Gathered Data

Data about age, gender, stroke risk factors (arterial hypertension, atrial fibrillation, diabetes mellitus, and dyslipidemia), stroke location, clinical severity, cortical damage, EEG changes, and occurrence of early and late ES after stroke were gathered.

The data were classified into groups:

- according to age, patients were divided into two groups: ≤65 years and >65 years;
- according to gender, patients were divided into males and females;
- according to stroke risk factors, patients were divided into having or not having arterial hypertension, atrial fibrillation, diabetes mellitus, and dyslipidemia;
- according to localization, acute ischemic strokes were divided into hemispheric, brainstem, or both;
- ischemic stroke severity was determined using the National Institutes of Health Stroke Scale (NIHSS) at admission. NIHSS was assessed for 294 (78.2%) patients. Based on stroke severity, patients were divided into 3 groups: NIHSS ≤ 3, NIHSS > 3 –< 11, and NIHSS ≥ 11. These NIHSS cut-off points were chosen as utilized in the SeLECT score [14];
- stroke treatment was divided into 4 groups: intravenous thrombolysis (IVT), mechanical thrombectomy (MTE), IVT and MTE, and no specific stroke treatment.
- according to radiological findings, patients were divided into 4 groups: acute cerebral cortical damage, acute cerebellar cortical damage, acute cerebral and cerebellar cortical damage, and subcortical damage. Since studies do not link ES to the damage of the cerebellar cortex, but to the cerebral cortex [24,25], we focused our calculations on the latter;
- EEG findings were divided into 2 groups and 2 subgroups: without focal changes, which was subdivided into normal and diffuse nonspecific changes (generalized slowing, generalized beta activity, etc.), and with focal changes, which was subdivided into focal slowing and interictal epileptiform discharges.

### 2.6. Data Analysis

Data were analyzed using the SPSS 27 software program. Sample-size normality was assessed using the Shapiro–Wilk test. Since no continuous variables followed a normal distribution, the means were compared using the Mann–Whitney U test. For categorical variables, Fisher’s exact test was used. To evaluate the probability of not developing late ES over time for patients with early ES, the Kaplan–Meier analysis was used. Statistical significance was assumed when *p* < 0.05.

## 3. Results

### 3.1. Demographic and Clinical Characteristics of Patients

From a total of 376 patients, 202 (53.7%) were male and 174 (46.3%) were female. The median age at stroke onset was 68 (25–94). There was a statistically significant difference between the median age of males (65 (26–94) and females (73 (25–92) (*p* < 0.001). According to age groups, 154 (40.7%) patients were ≤65 years and 223 (59.3%) were >65 years old.

The most common stroke risk factor was dyslipidemia (86.2%), followed by arterial hypertension (85.1%), atrial fibrillation (32.2%), and diabetes mellitus (19.4%). Hemispheric acute ischemic stroke was documented in 321 (85.4%), brainstem stroke in 53 (14.1%), and stroke in both locations in 2 (0.5%) patients. IVT was the most common treatment method (36.4%), while 33.5% did not receive specific stroke treatment, 17.8% received MTE, and 12.2% received IVT and MTE (Table 1).

### 3.2. Occurrence of ES

**Figure 1:**
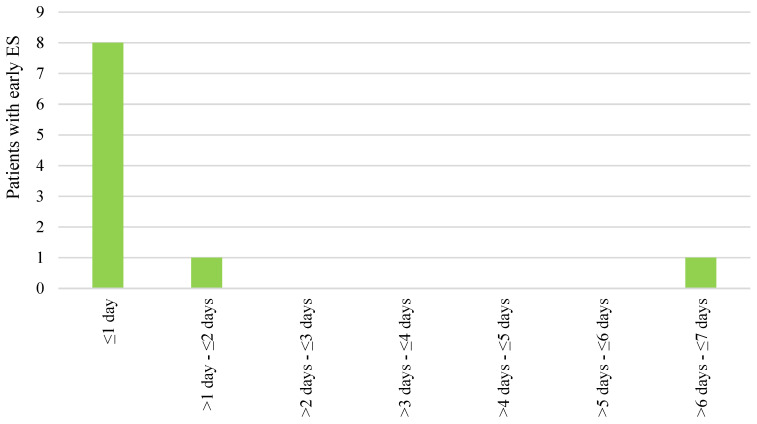
Occurrence of early epileptic seizures (ES) during the first seven days after ischemic stroke.

**Figure 2:**
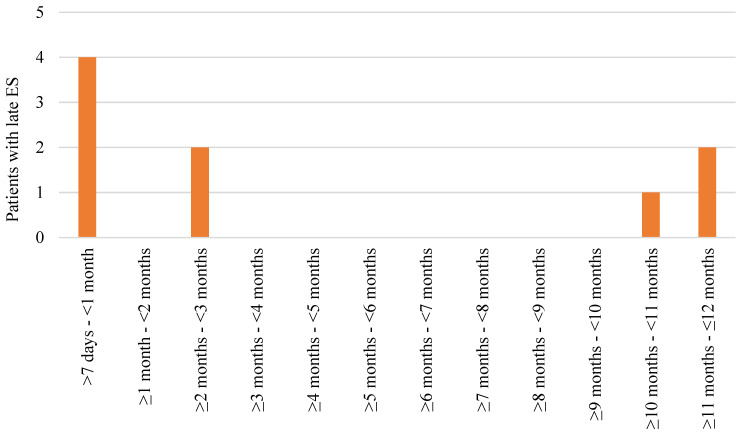
Occurrence of late epileptic seizures (ES) during the first year after ischemic stroke.

**Figure 3:**
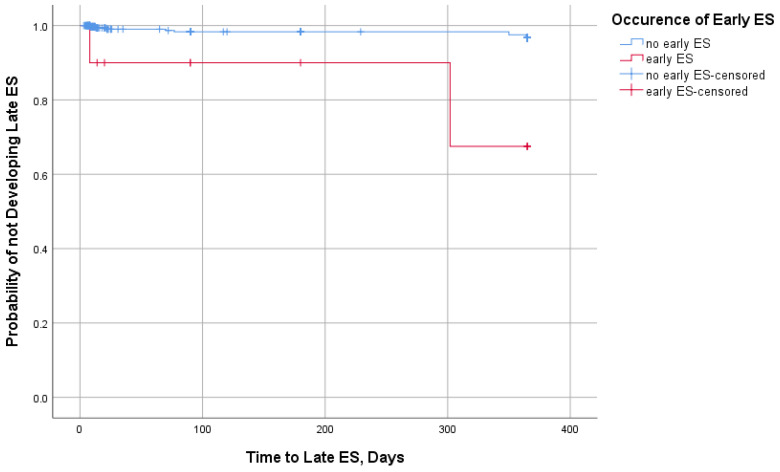
The probability of not developing late ES in patients with or without epileptic seizures (ES) (LogRank = 13.6, *p* < 0.001).

Out of 376 patients, 17 (4.5%) developed ES. Early ESs were recorded in 10 (2.7%) patients, with half of these cases coinciding with the onset of stroke (Figure 1). In total, nine (2.4%) patients experienced late ES, with the majority of cases occurring during the first month after stroke (Figure 2). Early ESs were either focal or focal to bilateral tonic–clonic in equal parts (five and five patients), while late ESs were more often focal to bilateral tonic–clonic than focal (seven and two patients, respectively). Two patients who had early ES also developed late ES. The occurrence of early ES increased the probability of developing late ES (LogRank = 13.6, *p* < 0.001) (Figure 3). All patients except one experienced a single early ES and were not prescribed antiepileptic drugs. One patient with recurrent early ES was put on 500 mg of levetiracetam twice daily; however late ES still developed. There was no statistically significant association of gender, age, stroke risk factors, location, severity, and treatment with the occurrence of ES (Table 1), early ES, or late ES.

### 3.3. Association of Acute Cortical Damage with the Occurrence of ES

Brain imaging detected acute cerebral cortical involvement in 51.3%, acute cerebellar cortical involvement in 6.6%, acute cerebral and cerebellar cortical involvement in 0.8%, and subcortical involvement in 41.2% of patients.

Cerebral cortical involvement was observed in 196 (52.1%) of our patients and was not documented in 180 (47.9%) patients. No statistically significant association between age, gender, arterial hypertension, diabetes mellitus, dyslipidemia, and stroke treatment with cerebral cortical damage was found. However, atrial fibrillation was more often present in patients with cerebral cortical damage (79 (40.3%) than in those without cerebral cortical damage (42 (23.3%) (*p* < 0.001). Also, patients more often had an NIHSS > 11 in cases with cerebral cortical damage (61 (39.6%) than those without (28 (20.1%) (*p* < 0.001).

When analyzing the association between cerebral cortical involvement with ES, early ES, and late ES, we found no statistically significant difference Table 2.

### 3.4. The Association of Electroencephalographic Changes and the Occurrence of ES

Out of 84 recorded EEGs, twenty (23.8%) were normal, 10 (11.9%) had diffuse nonspecific changes, 48 (57.1%) had focal slowing, and 6 (7.1%) had interictal epileptiform discharges. No subclinical ESs were registered on EEG. The time from stroke onset to EEG registration had no statistically significant impact on EEG findings (Table 3).

An EEG was recorded for 11 out of 17 patients with ES, 9 out of 10 patients with early ES, and 4 out of 9 patients with late ES. In patients with ES (*p* < 0.001), early (*p* < 0.001) or late ES (*p* = 0.024) interictal epileptiform discharges were found more often than focal slowing or normal and diffuse nonspecific changes (Table 4).

## 4. Discussion

In our study, 17 (4.5%) patients out of 376 developed ES. The incidence of early ES was 2.7%, while late ES occurred at 2.4%. This was consistent with the current literature that reports early ES rates after acute ischemic stroke ranging from 1% to 13.6% [8,32,33,34,35], and late ES from 2% to 9% [8,32,33,35]. Additionally, we also observed a similar increase in late ES risk after the occurrence of early ES [23,36,37]. An important fact to mention when discussing our findings is that the incidence of late ES in our study is closer to the lower end of the range, likely due to the short (1 year or shorter due to follow-up issues) observation period. While the risk of late ES peaks during the first year after stroke, it can manifest over a longer period of time. Hassani et al. described a median time of late ES in an acute ischemic stroke cohort of 237 days (interquartile range 33–688) [38]. In a study by Do et al., 55.7% of all late ESs occurred during the first year after acute ischemic stroke, 13.4% occurred during the second year, 7.0% during the third year, 5.5% during the fourth year, and 3.0% during the fifth year [36]. Although diminishing, seizure occurrence rates persist for around 10 years after acute ischemic stroke [36,39]. These findings suggest that a follow-up for more than 1 year would provide a more accurate estimation of the true incidence of late ES.

Though a few studies link younger age [16,17,18], male [40] or female [16] gender, atrial fibrillation [19,20], hypertension [21], stroke severity [16,19,41] and location [17,22] to an increased risk of ES, we did not find an association. It is worth mentioning that there was a statistically significant age difference between our male and female patients, which hindered an accurate comparison of the ES risk between different genders. However, it reflected the real world, since females experience acute ischemic strokes when they are approximately 4–6 years older than males [42,43].

A lot of authors associate cerebral cortical damage with an increased risk of ES [17,22,37,40,44]. Nandan et. al. carried out a systematic review and meta-analysis and found cortical involvement to be a risk factor for both early and late ES [24]. We did not find a link, possibly due to a low number of patients with ES. Subcortical damage is rarely considered a predictive factor. However, Lahti et al. discovered that the subcortical location of a primary intracranial hematoma is an independent risk factor for late ES [45]. To our knowledge, no similar association has been found with ischemic lesions with or without hemorrhagic transformation. Interestingly, while epilepsy is considered a cortical disease, subcortical regions may play a role in modulating epileptogenesis. This was hypothesized by Schaper et al. In a study, they mapped acute ischemic strokes in the brain and found that the epileptogenic lesion’s negative functional connectivity to a specific subcortical brain network, which includes the basal ganglia and cerebellum, is independently associated with ES and epilepsy [46].

According to our data, interictal epileptiform discharges were associated with the occurrence of ES, including both early and late ES. We noted that utilizing EEG findings to predict early ES is challenging due to ES concurrence with the onset of stroke, yet late ES association with focal-specific EEG changes was in agreement with the majority of published studies. By using a routine EEG during the first 72 h after stroke, Bentes et al. described interictal epileptiform activity as an independent predictor of post-stroke epilepsy [27]. Punia et al. used continuous EEG monitoring within the first week of stroke and found epileptiform activity to be more common in stroke patients than in healthy controls (58.3% versus 15.3%) and independently predictive of post-stroke epilepsy. The median time to first epileptiform discharge was 1.65 (interquartile range 0.3–10) hours after the start of EEG monitoring [26]. Other EEG findings have also been investigated. For example, Tatillo et al. described electrographic and electroclinical seizures; highly epileptogenic rhythmic and periodic patterns, such as lateralized periodic discharges; and regional attenuation without delta to be associated with post-stroke epilepsy [47]. On the other hand, in a subset of patients in a large matched multicenter study by Ferreira-Atuesta, EEG findings, such as diffuse or focal slowing, interictal epileptiform, or lateralized periodic discharges, were not independently predictive of post-stroke epilepsy [22]. We theorized that these contradictory findings might be in part explained by different methodologies; most researchers try to obtain an EEG within the first 72 h after stroke, while others try during the first week. However, we found no significant difference between EEG findings recorded within the first 72 h or after in our study population, and further research is required.

Our study is subject to several limitations. First, there was a small number of patients with ES, which might have led to inaccurate results and also hindered the performance of a multivariate analysis. Since only one patient with early ES was put on antiepileptic drugs, we could not perform a further analysis of the effect of antiepileptic drugs on the development of late ES. Second, the number of recorded EEGs was limited due to restricted equipment availability. It is important to note that the majority of our EEGs were registered for patients with strokes of mild or moderate severity due to the transportation difficulties of severely ill patients. Finally, patient follow-up lasted only for 1 year and, thus, might have resulted in a lower incidence of late ES.

Although our results were mostly in agreement with other reported findings, more research is clearly required. The acquisition of EEGs and the interpretation of EEG findings to predict increased ES risk still remain under-researched, with contradictory conclusions from various studies. More data is necessary to explore the significance of age, gender, stroke risk factors, severity, location, cortical involvement, or various combinations of these risk factors for developing ES after acute ischemic stroke.

## 5. Conclusions

In our study, the incidence of ES was 4.5%, the incidence of early ES was 2.7%, and the incidence of late ES was 2.4%. The occurrence of early ES increased the probability of developing late ES. There was no association between gender, age, stroke risk factors, location, severity, acute cerebral cortical damage, and stroke treatment with the occurrence of ES, including both early and late ES. Interictal epileptiform discharges were associated with the occurrence of ES, including both early and late ES.

## Figures and Tables

**Table 1:** Demographic and clinical characteristics of patients.

Demographic and Clinical Characteristics	All Patients (n = 376)	Patients with ES (n = 17)	Patients without ES (n = 359)
Gender			
Male, n (%)	202 (53.7)	8 (47.1)	194 (54.0)
Female, n (%)	174 (46.3)	9 (52.9)	165 (46.0)
Age (years), median (range)	68 (25–94)	69 (43–88)	68 (43–88)
Males	65 (26–94)	68.5 (43–88)	65 (26–94)
Females	73 (25–92) *	74 (47–86)	73 (25–92) *
Age Groups			
≤65 years, n (%)	153 (40.7)	6 (35.3)	147 (40.9)
>65 years, n (%)	223 (59.3)	11 (64.7)	212 (59.1)
Stroke Risk Factors			
Arterial Hypertension, n (%)	320 (85.1)	15 (88.2)	305 (85.0)
Atrial Fibrillation, n (%)	121 (32.2)	6 (35.3)	115 (32.0)
Diabetes Mellitus, n (%)	73 (19.4)	5 (29.4)	68 (18.9)
Dyslipidemia, n (%)	324 (86.2)	14 (82.4)	310 (86.6)
Stroke Location			
Hemispheric, n (%)	321 (85.4)	14 (82.4)	307 (85.5)
Brainstem, n (%)	53 (14.1)	3 (17.6)	50 (13.9)
Both, n (%)	2 (0.5)	0 (0)	2 (0.6)
Stroke Severity (n = 293)			
NIHSS ≤ 3, n (%)	62 (21.1)	2 (15.4)	60 (21.4)
NIHSS > 3–<11, n (%)	142 (48.5)	4 (30.8)	138 (49.3)
NIHSS ≥ 11, n (%)	89 (30.4)	7 (53.8)	82 (29.3)
Type of Stroke Treatment			
IVT, n (%)	137 (36.4)	8 (47.1)	135 (36.8)
MTE, n (%)	67 (17.8)	3 (17.6)	65 (17.7)
IVT and MTE, n (%)	46 (12.2)	2 (11.8)	44 (12.0)
No Specific Treatment, n (%)	126 (33.5)	4 (23.5)	123 (33.5)

* *p* < 0.05, Mann–Whitney U test—compared to males; IVT—intravenous thrombolysis, MTE—mechanical thrombectomy, NIHSS—National Institutes of Health Stroke Scale.

**Table 2:** The association of acute cerebral cortical involvement and the occurrence of epileptic seizures (ES).

	Patients with Acute Cerebral Cortical Damage (n = 196)	Patients without Acute Cerebral Cortical Damage (n = 180)
No ES, n (%)	186 (94.9)	173 (97.1)
ES, n (%)	10 (5.1)	7 (2.9)
Early ES, n (%)	4 (2.0)	6 (3.3)
Late ES, n (%)	7 (3.6)	2 (1.1)

*p* > 0.05, Fisher’s exact test; ES—epileptic seizures.

**Table 3:** The association of electroencephalographic (EEG) changes and time from stroke to EEG registration.

EEG Changes	Time from Stroke to EEG Registration	
	≤3 Days (n = 62)	>3 Days (n = 22)
Normal EEG, n (%) Diffuse Nonspecific Changes, n (%)	16 (25.8) 7 (11.3)	4 (18.2) 3 (13.6)
Focal Slowing, n (%) Interictal Epileptiform Discharges, n (%)	36 (58.1) 3 (4.8)	12 (54.5) 3 (13.6)

*p* > 0.05, Fisher’s exact test; EEG—electroencephalogram.

**Table 4:** The association of electroencephalographic (EEG) changes and the occurrence of epileptic seizures (ES).

	No Focal EEG Changes	Focal EEG Changes	
	Normal and Diffuse Nonspecific (n = 30)	Focal Slowing (n = 48)	Interictal Epileptiform Discharges (n = 6)
No ES, n (%)	28 (93.3)	44 (91.7)	1 (16.7) *
ES, n (%)	2 (6.7)	4 (8.3)	5 (83.3) *
Early ES, n (%)	1 (3.3)	4 (8.3)	4 (66.7) *
Late ES, n (%)	1 (3.3)	1 (2.1)	2 (33.3) *

* *p* < 0.05, Fisher’s exact test—compared to no focal EEG changes and focal nonspecific changes; EEG—electroencephalogram, ES—epileptic seizures.

## Data Availability

Data are contained within the article.

## Abbreviations

CT—computed tomography, EEG—electroencephalogram, ES—epileptic seizures, IVT—intravenous thrombolysis, MRI—magnetic resonance imaging, MTE—mechanical thrombectomy, NIHSS—National Institutes of Health Stroke Scale.
